# Adoptive transfer of splenocytes to study cell-mediated immune responses in hepatitis C infection using HCV transgenic mice

**DOI:** 10.1186/1476-5926-9-7

**Published:** 2010-08-20

**Authors:** Turaya Naas, Masoud Ghorbani, Catalina Soare, Nicole Scherling, Rudy Muller, Peyman Ghorbani, Francisco Diaz-Mitoma

**Affiliations:** 1 Infectious Disease and Vaccine Research Centre, Children's Hospital of Eastern Ontario Research Institute, Ottawa, ON, Canada; 2 Department of Microbiology, Immunology and Biochemistry, University of Ottawa, Ottawa, ON, Canada; 3 Division of Virology, Children's Hospital of Eastern Ontario, Ottawa, ON, Canada; 4 Health Canada, Ottawa, ON, Canada; 5 Production and Research Complex of Pasteur Institute of Iran, Tehran-Karaj, I.R. Iran

## Abstract

**Background:**

Hepatitis C virus (HCV) is a major cause of chronic hepatitis and a health problem affecting over 170 million people around the world. We previously studied transgenic mice that express HCV Core, Envelope 1 and Envelope 2 proteins predominantly in the liver, resulting in steatosis, liver and lymphoid tumors, and hepatocellular carcinoma. Herein, the immune-mediated cell response to hepatitis C antigens was evaluated by adoptive transfers of carboxyfluorescein succinimidyl ester (CFSE) labelled splenocytes from HCV immunized mice into HCV transgenic mice.

**Results:**

In comparison to non-transgenic mice, there was a significant decrease in the percentage of CFSE-labeled CD4^+ ^and CD8^+ ^T cells in transgenic mouse peripheral blood receiving adoptive transfers from immunized donors. Moreover, the percentage of CFSE-labeled CD4^+ ^and CD8^+ ^T cells were significantly higher in the spleen of transgenic and non-transgenic mice when they received splenocytes from non-immunized than from immunized mice. On the other hand, the percentages of CD4^+ ^and CD8^+ ^T cells in the non-transgenic recipient mouse lymph nodes were significantly higher than the transgenic mice when they received the adoptive transfer from immunized donors. Interestingly, livers of transgenic mice that received transfers from immunized mice had a significantly higher percentage of CFSE labeled T cells than livers of non-transgenic mice receiving non-immunized transfers.

**Conclusions:**

These results suggest that the T cells from HCV immunized mice recognize the HCV proteins in the liver of the transgenic mouse model and homed to the HCV antigen expression sites. We propose using this model system to study active T cell responses in HCV infection.

## Introduction

Hepatitis C virus (HCV) is a major cause of chronic liver disease worldwide. The virus causes chronic infection in 80% of acutely HCV-infected patients; a subset of these individuals develop progressive liver injury leading to liver cirrhosis and/or hepatocellular carcinoma [1,2]. Immune responses to HCV play important roles at various stages of the infection. There is emerging evidence that the ability of acutely HCV-infected patients to control the primary HCV infection depends on the vigorous cellular immune reaction to the virus [3]. In the chronic phase of infection, immune responses determine the rate of progression of disease, both by limiting viral replication and by contributing to immunopathology. Livers from chronically HCV-infected individuals show T cell infiltration; however, these cells are not HCV specific and are unable to eradicate the virus [4]. These liver-infiltrating lymphocytes are associated with liver damage in chronic HCV infection via mechanisms that are not well understood [5]. There are several immune evasion mechanisms, which might explain the ability of the virus to escape the immune responses and establish a persistent infection. These immune evasion strategies include: virus mutation, primary T cell response failure, impairment of antigen presentation, suppression of T cell function by HCV proteins, impairment of T cell maturation and a tolerogenic environment in the liver [6]. Nevertheless, the immunological basis for the inefficiency of the cellular immune response in chronically infected persons is not well understood.

Cellular immune responses play a critical role in liver damage during the clinical course of hepatitis C infection. HCV-specific CD4^+ ^T cells are involved in eradication of the virus in acute infection but their responses are weak and insufficient in chronic hepatitis [7]. However, there is no clear evidence that CD4^+ ^T cells play a direct role in the liver injury observed during chronic HCV infection. CD4^+ ^T cells activate the CD8^+ ^cytotoxic T lymphocyte (CTL) response, which eradicates the virus-infected cells either by inducing apoptosis (cytolytic mechanism) or by producing interferon-gamma (IFN-γ), which suppresses the viral replication (non-cytolytic mechanism) [8]. Enhanced hepatocyte apoptosis leads to liver damage in chronic HCV infections [9]. HCV-specific CD8^+ ^CTL responses are compromised in most patients who fail to clear the infection. In addition, those cells have a diminished capacity to proliferate and produce less IFN-γ in response to HCV antigens [10]. Those inefficient CD8^+ ^T cell responses mediate HCV-related liver damage and are inadequate at clearing the chronic infection.

The mechanisms responsible for immune-mediated liver damage associated with HCV are poorly understood. One of the mechanisms for liver damage is that the HCV-activated T cells express the Fas ligand at the cell surface, which will bind with the Fas receptor on hepatocytes, initiatiating Fas-mediated signaling, which may then lead to cell death [11]. HCV core protein increases the expression of Fas ligand on the surface of liver-infiltrating T cells leading to the induction of hepatic inflammation and liver damage [12,13]. Another important mechanism of immune-mediated liver damage is through CD8^+ ^T cell-mediated cytolysis. Previous studies on concanavalin-A-induced hepatitis have demonstrated that CD8^+ ^T cells can kill the target cells *in vivo *by cytolytic mechanisms mediated by perforin [14] or requiring IFN-γ [15]. This may also involve additional molecules such as TNF-α [16]; therefore, the level of cytolytic activity or expression of cytolysis mediators from the infiltrating lymphocytes could be a determinant for induction of immune-mediated liver damage.

It is still controversial whether the liver damage associated with hepatitis C infection is due to the viral cytopathic effects or due to the immune response mediated damage. Previously, we demonstrated the direct effect of viral proteins in the pathogenesis of HCV infection by developing a HCV transgenic mouse model that expressed the HCV structural proteins, Core, E1 and E2 predominantly in the liver [17]. This model showed hepatopathy, including hepatic steatosis and liver tumors. In this study, we describe a model to examine immune-mediated liver cell damage by means of adoptive transfer of splenocytes from HCV immunized mice into HCV transgenic mice. Our results showed that the carboxyfluorescein succinimidyl ester (CFSE)-labeled T cells from HCV immunized mice homed to the liver of HCV transgenic mice, indicating that these HCV-activated T cells recognize the HCV transgene and attack the hepatocytes expressing it, which may lead to liver damage.

## Methods

### Mice

All mice used in the study were purchased from the Charles River Laboratories (Senneville, QC, Canada) and were from a B6C 3F1 genetic background. Mice were bred in specific pathogen-free conditions at the animal care facilities at the University of Ottawa. Animals were used according to the guidelines of the animal care committee at the University of Ottawa. Donor mice were 6 to 8 weeks old; wild type mice and the recipient mice, both HCV transgenic and non-transgenic mice, were 3 to 6 months old. The establishment and characterization of these HCV transgenic mice were described in our previous study [17].

### Plasmids and proteins

Construction of pVAX Core, E1 and E2 expression vector was described in our previous study [17]. Briefly, total RNA extracted from the plasma of a patient infected with HCV genotype 1a was used as a template to amplify Core, E1, and E2 genes. The HCV fragment containing Core, E1, and truncated E2 genes was constructed through RT-PCR using forward primer 5' ACC ATG AGC ACG AAT CCT AAA CCTC 3' and reverse primer 5' TGG TAG GGT TGT GAA GGA ACA CG 3'. The amplified fragment was cloned into the EcoR1 sites of pCR 2.1 vector using the TOPO-TA cloning kit (Invitrogen, Burlington, ON). The nucleotide sequence was verified by DNA sequencing using the University of Ottawa DNA sequencing facility. The Core, E1, E2 fragment was subsequently subcloned into pVAX-1 plasmid (Invitrogen, Burlington, ON) downstream of a cytomegalovirus promoter. The expression vector of recombinant HCV Core, E1 and E2 polyprotein was also described in our previous study [18]. Briefly, the TOPO-TA HCVcore/E1/E2 construct was subcloned into the pEF6/Myc-His expression vector (Invitrogen Burlington, ON); this vector contains six histidine residues which permit purification of the HCV polyprotein by immobilized metal affinity chromatography (Clontech Talon Metal Affinity Resin Kit, Palo Alto, CA). The recombinant plasmid containing the correctly oriented insert was transfected into DH5 cells, amplified, and purified using the Endofree plasmid purification kit (Qiagen), as previously described. Chinese hamster ovary cells were transiently transfected with the recombinant pEF6/Myc-His vector containing the core/E1/E2 insert. Transfection was performed by 2 electroporation shocks at 1.4-1.6 KV using an electroporation apparatus (BTX Inc., San Diego, CA). The transfected cells were incubated in IMDM (Sigma-Aldrich, St. Louis, MO) containing 10% FCS (Life Technologies Laboratories, Grand Island, NY) and 50 *μ*g/mL penicillin-gentamicin. At 65 hrs after transfection the cells were harvested, lysed in lysis buffer (25 mmol/L Tris base, 2.5 mmol/L mercaptoethanol, and 1% Triton-X100), sonicated, and subjected to protein purification using the Talon affinity resin kit as described before. The purity of the protein was verified by mass spectrometry, and protein with ~85% purity was used for immunization.

### Immunization strategy of donor mice

Eight donor mice were immunized with a HCV vaccine containing pVAX-HCV Core, E1 and E2 DNA (100 μg); Core, E1 and E2 protein (25 μg) in PBS solution and montanide (50 μl) ISA-51 (Seppic Inc., Fairfield, NJ) was used as adjuvant. Mice were immunized three times with 100 μl of the vaccine and boosted twice intramuscularly in the quadriceps major with two weeks intervals between each boost. Eight wild-type non-immunized mice were injected with PBS solution and montanide ISA-51 alone and used as a negative control. After each immunization, the humoral immune response was assessed by an IgG ELISA using mouse sera. The cellular immune response was assessed using PBMCs isolated from the whole blood after the first immunizations and using PBMCs isolated from splenocytes after the last immunization. The mice were anesthetized with 50 Somnotal (MTC Pharmaceuticals, Cambridge, ON, Canada), sacrificed, and blood and spleens were collected.

### Preparation of lymphocytes from donor mouse spleens

Donor mice were sacrificed using anesthetic, and spleens were removed and placed in tubes containing sterile PBS. Lymphocytes were prepared as a cell suspension by gently pressing organ segments through a fine plastic cell strainer using a plastic pipette; then, 10 ml of PBS was added to pass cells through the mesh. The spleen cell suspensions were depleted of red blood cells (RBC) using RBCs lysis buffer (155 mM NH_4_Cl, 10 mM KHCO_3_, and 0.1 mM EDTA). The cellular suspension was washed three times by adding 0.1% BSA in PBS and centrifuged at 1600 rpm at 4°C for 5 min. The cells were counted and divided into 2 parts: cells for CFSE labeling, which were used for injection and CFSE proliferation assay, and cells for CTL and ELISPOT assays used to assess the immune response.

### ELISA

To assess the antibody titer against the HCV vaccine, mice were bled at different points after the immunizations and the serum was collected. Serum levels of hepatitis C-specific antibodies were measured using the HCV recombinant core/E1/E2 polyprotein as a capture molecule and a mouse-specific monoclonal antibody-horseradish peroxidase (HRP) conjugate detection system. EIA/RIA Stripwell™ plates (Corning CoStar Inc., New York, NY) were coated with 20 μg/ml recombinant core/E1/E2 poly protein dissolved in sterile distilled/deionized water for 4 hrs and incubated overnight at 4°C. After washing, the plates were blocked with 1% BSA (Sigma-Aldrich, St. Louis, MO) in PBS for 1 hr at 37°C. Then the plates were washed and dilutions of sera were incubated for 2 hrs at 37°C. Antibodies were detected with a 1/1000 dilution in 1% BSA/PBS of the required goat anti-species-specific HRP conjugate (IgG H+L: Jackson Immunoresearch Laboratories, West Grove, PA; IgG1, IgG2a: Serotec, Oxford, UK). After each incubation time, the plates were washed six times with PBS/0.05% Tween-20 (Sigma-Aldrich). O-phenylenediamine dihydrochloride (Sigma-Aldrich) and hydrogen peroxide were used to develop the color reaction. The optical density (OD) was read at 490 nm after the reaction was stopped with 1 N HCl. An IgG2a monoclonal antibody specific for core protein amino acids 1-120 (Clone 0126, Biogenesis Ltd., Poole, England) and hepatitis C-negative or pre-immune sera were run in parallel with all samples tested as negative control. OD values of at least 2 standard deviations above the mean OD from the pre-immunization sera were considered positive for an HCV-antibody response.

### IFN-γ intracellular staining

CD8^+ ^CTL responses were assessed by measuring the mouse IFN-γ production using intracellular staining. The intracellular procedures were done according to Caltag Laboratories protocol. Briefly, PBMCs isolated from fresh blood or the splenocytes of immunized mice were cultured in complete RPMI media in the presence of 10 μg/ml brefeldin A (Sigma) and stimulated with core, E1 and E2 protein, core peptides, or vaccinia poly HCV (NIH AIDS, Cat# 9426) expressing HCV-1 Core, E1, E2, P7 and NS2 truncated. Unstimulated or empty vaccinia stimulated cells were used as a negative control. PMA/ION stimulated cells were used as a positive control. Eighteen hrs after incubation at 37°C, the cells were washed with PBS/2% FCS/0.01% sodium azide and surface-stained for 15 min with PE-labeled monoclonal antibody against mouse CD3^+^, TC-labeled antibody to mouse CD8^+ ^or CD4^+ ^(Caltag Laboratories, Hornby, ON). The cells were washed as above, fixed and permeabilized using Caltag reagent A and B fixation-permeabilization solutions (Caltag Laboratories). The cells were stained intracellularly with anti-mouse IFN-γ FITC-labeled Ab and incubated for 30 min (in the dark) at 4°C. Following washing, cells were analyzed in a FacScan flow cytometer (Becton Dickinson, Mississauga, ON). An increase of 0.1% of IFN-γ producing cells over the unstimulated control or empty vaccinia virus stimulated cells were considered as positive response to vaccination.

### IFN-γ ELISPOT

The ELISPOT assay was performed according to Mabtech protocol. Briefly, a 96-well microtiter plate was coated with mouse anti-IFN-γ monoclonal antibodies (10 μg/ml in PBS). The cells (250,000/well) were added to the plate with cross bonding stimulants. Cells stimulated with core, E1 and E2 protein, core peptides, or vaccinia poly HCV. Unstimulated or empty vaccinia stimulated cells were used as a negative control. PMA/ION stimulated cells were used a positive control. After 48 hrs of incubation, the cells were removed by washing and a biotinylated antibody against IFN-γ (10 μg/ml in PBS) was added. In the subsequent, the streptavidin conjugated with enzyme ALP was added. Finally, a precipitation substrate (BCIP) for ALP was added and the plates were incubated until spots emerged at the site of the responding cells. The spots were examined and counted in an image analyzer system. The mean number of specific spot-forming cells (SFCs) was calculated by subtracting the mean number of spots from unstimulated cells or empty vaccinia stimulated cells from the mean number of spots in cells stimulated with core, E1 and E2 or core peptides or recombinant HCV poly vaccinia.

### Lymphocytes proliferation assay

The CD4^+ ^T cell proliferation was assessed after labeling the lymphocytes derived from the spleen using CFSE dye (Invitrogen Molecular Probes).

### Labeling cells with CFSE

Ten mM of CFSE stock solution was prepared by adding 90 μl Dimethyl Sulfoxide (DMSO) to 500 μg lyophilized powder of CFSE dye. The stock solution was diluted in sterile PBS/0.1% BSA to get the desired working concentration of 10 μM. Purified lymphocytes were resuspended to a concentration of 50 million cells per ml in PBS/0.1% BSA before the addition of CFSE dye. An equal volume of 10 μM of CFSE dye was added to the cell suspension in a tube 6 times more than the volume of the cell suspension and mixed well by vortexing. The labeled lymphocytes were incubated for 15 min at 37°C. The staining was quenched by adding 5 volumes ice-cold complete RPMI media followed by a 5 min incubation on ice. The cells were washed three times in complete RPMI media and re-suspended in complete RPMI (2 million cells per ml for the proliferation assay and 40 million cells in 75 μl PBS for injecting to mice). To verify the CFSE-labeled cells, samples of the cell suspensions were run on a flow cytometer and were also analyzed by fluorescent microscopy. The proliferation was assessed after stimulation of the cells with core, E1 and E2 proteins (10 μg/ml) or core peptides (10 μg/ml). PMA (10 ng/ml) and ionomycine (1 μg/ml) were added to the cells as a positive control. After adding the stimulant, the cells were incubated at 37° in 5% CO_2 _for 4 days. The stimulated cells were then harvested by centrifugation at 1600 rpm for 5 min. The prodedures for statining and manipulation of CFSE labeled cells should be done in the dark.

### Surface stain each stimulated cell with CD3 TC and CD4 PE for 3 colour flow cytometry

The cells were incubated 15 min in the dark at room temperature. After washing with PBS/0.1 azide/5% FCS, the cells were immediately analyzed on FacScan or were fixed by adding an equal volume of 2% paraformaldehyde and stored overnight at 4°C before the analysis. Cells stained with CFSE have very bright fluorescence. As the cells proliferate, the fluorescence of the cell populations decreases from bright to dim. Daughter cells have half the fluorescent intensity of the parent cell.

### Injection of labeled cells into recipient mice

CFSE labeled cells from the donor mice (n = 7) were pooled and injected through the tail veins of the recipient mice (n = 7). Twenty million cells suspended in 75 μl of PBS per mouse were injected. The mice were bled 24 hrs after the injection and then sacrificed 7 days later. The following tissues were collected and processed for further analysis: blood, lymph nodes, spleen, thymus and liver.

### Flow cytometry

The tissues were processed to get cell suspensions by gently pressing the tissue through the cell strainer and collecting the cells in sterile PBS. The RBCs were lysed from the blood (3-4 times), spleen and lymph nodes (1 time). The cells were counted and alliquoted and surface stained with fluorescence-labelled antibodies directed at mouse CD3^+^, CD4^+^, or CD8^+ ^for differentiation. Flow cytometry was carried out on a 4-color flow cytometry instrument (CEPICS XL Flow Cytometry Systems, Beckman Coulter, Inc). Instrument settings were adjusted so that fluorescence of cells from non-immunized controls or negative controls fell within the first decade of a four decade logarithmic scale on which emission is displayed. Flow cytometry plots showed at least 20,000 events. The data were analyzed by FlowJo software (Tree Star Inc., Ashland, Oregon) in accordance with the manufacturer instructions. The expression levels of different surface antigen markers as well as an intracellular proliferating marker were analyzed.

### Fluorescence microscopy

Fluorescence microscopy was used to locate lymphocytes in intact organs. One to two mm thick sections of fresh frozen liver and spleen were mounted in mounting media in a recessed microscope slide and examined under fluorescence microscopy (excitation at 491 nm and emission at 518 nm).

### Histological analysis

To study the histological changes, mouse livers were fixed in 4% paraformaldehyde and embedded in paraffin. Five μm thick sections were stained with hematoxylin and eosin (H&E) according to standard methods used in the Department of Pathology and Laboratory Medicine at the Faculty of Medicine, University of Ottawa.

### Statistical data analysis

Statistical analysis used Instat software to do an ANOVA, followed by Student-Newman-Keuls post hoc test. Significant differences are based on *P *< 0.05.

## Results

### Immune response in HCV-immunized donor mice

**Figure 1:**
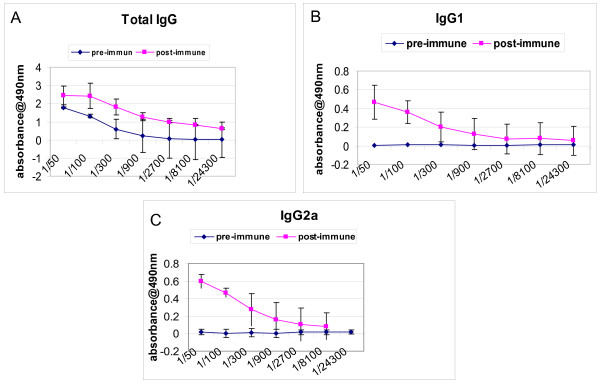
**Humoral immune responses of the donor mice immunized with HCV immunogens as determined by ELISA**. Seven mice were immunized with HCV immunogens containing HCV plasmid DNA, HCV recombinant polyprotein and montanide. Mice were immunized three times intramuscularly and boosted twice with the same vaccine. After the third immunization, serum samples were collected, serially diluted and tested for reactivity with HCV core, E1 and E2 protein. Sera were collected from the mice pre-immunization were used as a baseline. Immunized mice had significant increase in total IgG, IgG1, and IgG2a after the third immunization at 1:900 antibody titer (* *P *< 0.05).

**Figure 2:**
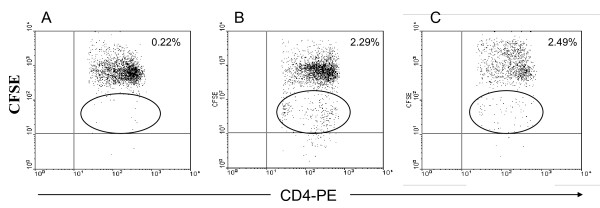
**CD4^+ ^T cell proliferation response of HCV-immunized mice**. The splenocytes were stained with CFSE dye and incubated with different stimulants for 4 days. Cells were stained for surface markers using anti-CD3^+ ^and CD4^+^-antibodies and tested using flow cytometry. (A) Unstimulated cells showing no proliferation, (B) CE1E2 protein-stimulated cells showing proliferation of the cells which is indicated by the shift of fluoresecence in the cell population (circle), (C) Core peptide stimulated cells showing proliferation. Daughter cells contain half the fluorescent intensity of the parent cell.

**Figure 3:**
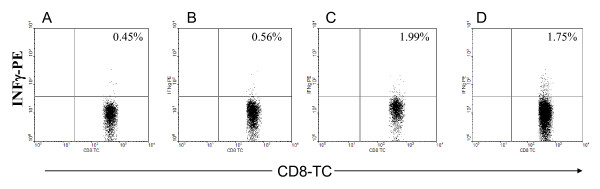
**CD8^+ ^T cells cytolytic activity in the immunized mice as demonstrated by IFN-γ intracellular staining**. Two weeks after the last HCV vaccine immunization, cultured splenocytes were unstimulated (A), stimulated with CE1E2 protein (B), core peptide (C), or vaccinia HCV poly (D). Cells were cultured for 18 hrs in the presence of brefeldin A then stained intracellularly with anti-IFN-γ antibody and surface stained with anti-CD3^+ ^and anti-CD8^+ ^antibodies to be analyzed by flow cytometry. Percentages in the upper right quadrant represent the frequency of CD3^+^8^+ ^T lymphocytes expressing IFN-γ. The *P *value for significant differences was < 0.05.

**Figure 4:**
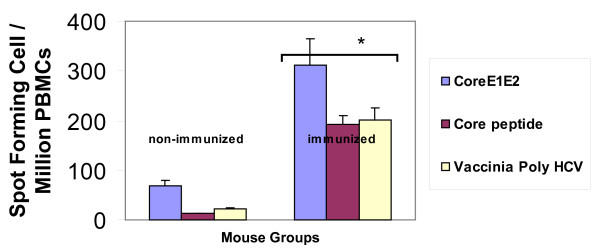
**Detection of CD4^+ ^and CD8^+ ^T lymphocyte responses to HCV vaccine in immunized mice using IFN-γ ELISPOT assay**. ELISPOT counts (spot-forming units [SFUs]/1 × 10^6^) in response to core, E1 and E2 protein, Core peptides, or vaccinia HCV poly. Spot forming cell (SFC) frequencies are shown after subtraction of background with unstimulated cells or empty vaccinia stimulated cells. Cells were incubated with core, E1 and E2 protein, Core peptides, or vaccinia HCV poly for 48 hrs before measuring IFN-γ ELISPOT responses. Spot forming cell (SFC) frequency per million cells is indicated for each immunized and non-immunized donor mice. The *P *value was < 0.05.

We developed a hepatitis C transgenic mouse model in which the HCV structural proteins are predominantly expressed in the liver [17]. We used this model to analyze the kinetics of immune cells featuring an antiviral immune response against hepatitis C in adoptive transfer experiments after immunization with an HCV vaccine candidate. Previously, we showed that mice immunized with a combinations of a candidate HCV vaccine consisting of recombinant HCV core/E1/E2 DNA plasmid and rHCV polyprotein and montanide demonstrated significant humoral and cellular immune response [18]. In this study, we used the same strategy to immunize the donor mice. Mice immunized with a combined HCV vaccine consisting of both HCVcore/E1/E2 DNA and protein and the adjuvant montanide A51 showed humoral and cellular antiviral immune responses. The ELISA assay demonstrated a significant increase in the antibody titer against HCV immunogens. There was a significant increase in total IgG, IgG1, and IgG2a after the third immunization at 1:900 antibody titer (* *P *< 0.005) (Figure 1). Similarly, in response to HCV antigens CD4^+ ^T cell proliferation was demonstrated by CFSE staining. After the last immunization the splenocytes were cultured in the presence of core, E1 and E2 polyprotein or core peptides. There was a marked increase in the proliferation response of the immunized mouse splenocytes when they were stimulated with HCV Core/E1/E2 or core peptides, as indicated by the decrease in the CFSE stain intensity. As the cells proliferate, the cell population shifts to a lower intensity due to the decrease of staining in the cell membranes of proliferating cells. Daughter cells have half the fluorescent intensity of the parent cells (Figure 2). CD8^+ ^T cell cytolytic activity was demonstrated by INF-γ production using intracellular staining and ELISPOT. INF-γ production was significantly higher in immunized mice compared to controls (Figure 3, 4). Approximately 2% of the CD8^+ ^T cells produced IFN-γ when they were stimulated with HCV core peptide and 1.75% of the cells produced IFN-γ when they stimulated with vaccinia encoding HCV recombinant proteins (vaccinia HCV poly) (Figure 3c, d). These results were confirmed by IFN-γ ELISPOT. It indicated that splenocytes from immunized mice produced significantly more IFN-γ when they were stimulated with core, E1 and E2 protein, core peptides or vaccinia encoding HCV recombinant proteins (vaccinia HCV poly) (*P *< 0.05) (Figure 4).

### Flow cytometric analysis of recipient mouse tissues

**Figure 5:**
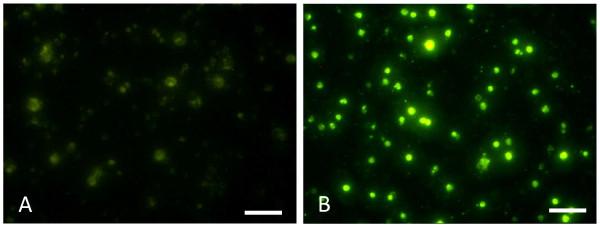
**Immunofluoresent analysis of CFSE labeled splenocytes before injection**. A) CFSE unlabeled splenocytes showing no CFSE staining. B) CFSE labeled splenocytes showing green fluorescent cells. Scale bar = 50 μm.

To study the splenocyte kinetics in the HCV transgenic mice and to indirectly evaluate the immune response generated after HCV vaccination, splenocytes from the immunized and control mice were collected and labeled with CFSE before performing the adoptive transfer. CFSE labeled splenocytes were then confirmed by immunofluorescent microscopy (Figure 5). These cells were injected intravenously in transgenic and control mice and tracked down in the blood *in vivo *after 24 hrs. Seven days after the adoptive transfer, recipient mice were euthanized. The location and number of transferred cells were detected by flow cytometry in blood, lymph nodes, spleens and livers of recipient mice.

**Figure 6:**
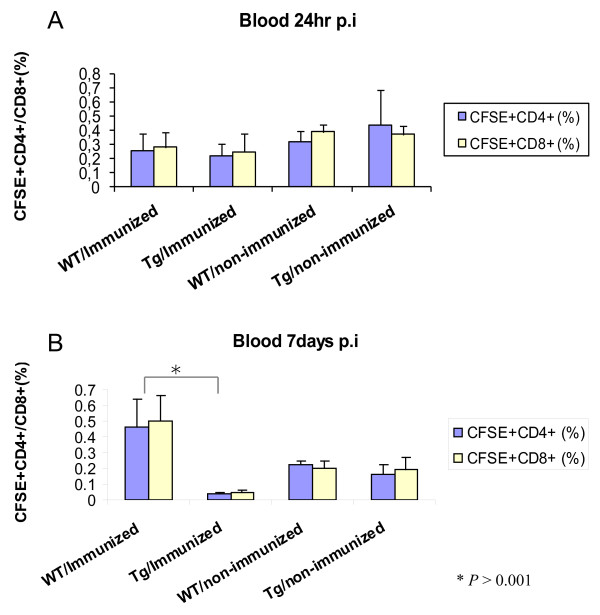
**Flow cytometric analysis of recipient mouse blood 24 hrs and 7 days post-adoptive transfer**. A) The percentage of CFSE CD4^+ ^and CD8^+ ^T cells in the blood of the recipient mice 24 hrs post-injection. The × axis indicates the donor and recipient mouse groups (n = 7) and the Y axis indicate the percentage of the CFSE^+ ^CD4^+ ^or CD8^+ ^T cells B) The percentage of donor CD4^+ ^and CD8^+ ^T cells in the blood seven days after the injection. The cells were surface stained with anti-CD3^+ ^and anti-CD4^+ ^antibodies or anti-CD3^+ ^and anti-CD8^+ ^and analyzed by flow cytometry (*P <*0.001).

All groups of recipient mice had similar percentages of donor CD4^+ ^and CD8^+ ^T cells at 24 hrs post-adoptive transfer, indicating that all groups received similar amounts of donor splenocytes (Figure 6a). Seven days after the adoptive transfer, the percentage of the donor CD4^+ ^and CD8^+ ^T cells in the blood differed between the recipient mice receiving immunized and non-immunized donor cells (Figure 6b). There was a significant increase in the percentage of donor T cells in the blood of wild type mice receiving immunized donor cells. In contrast, there was a significant decrease in the percentage of donor T cells in the blood of transgenic mice having received immunized donor cells. In fact, among the groups of mice studied, the transgenic animals had the lowest percentage of donor T cells in the blood (Figure 6b). There was no significant difference of donor cell percentages in the groups receiving cells from non-immunized donors.

**Figure 7:**
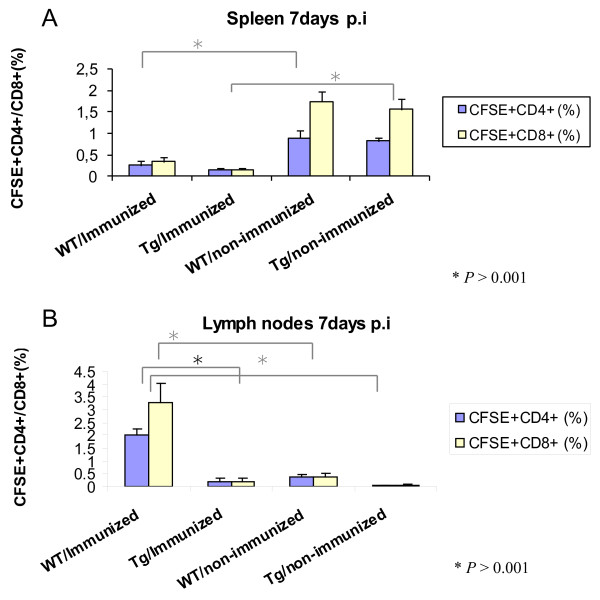
**Flow cytometric analysis of recipient mouse spleens and lymph nodes**. A) The percentage of CD4^+ ^and CD8^+ ^T cells in the spleens of mice receiving immunized and non- immunized donor cells. B) The percentage of CD4^+ ^and CD8^+ ^T cells in the lymph nodes of the recipient mice. The cells were surface stained with anti-CD3^+ ^and anti-CD4^+ ^antibodies or anti-CD3^+ ^and anti-CD8^+ ^and analyzed by flow cytometry (*P <*0.001).

**Figure 8:**
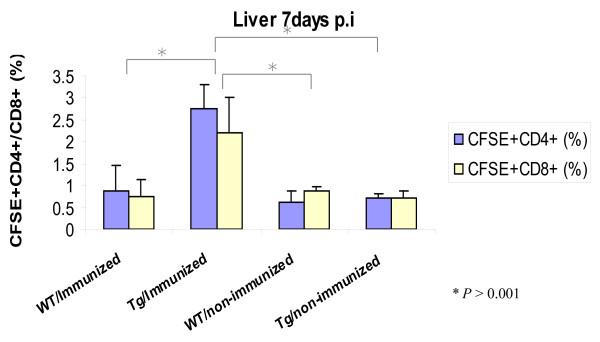
**Flow cytometric analysis of recipient mouse livers**. The percentage of CD4^+ ^and CD8^+ ^T cells in the liver of mice receiving immunized and non-immunized donor cells was detected by FACS. The cells were surface stained with anti-CD3^+ ^and anti-CD4^+ ^antibodies or anti-CD3^+ ^and anti-CD8^+ ^and analyzed by flow cytometry (*P <*0.001).

A higher percentage of donor T-cells from the non-immunized groups homed to the spleen as compared to the immunized animals. There was a four to ten-fold increase in the number of CD4^+ ^and CD8^+ ^T cells in the spleens of mice receiving non-immunized donor (Figure 7a). The donor cells from immunized animals homed to the lymph nodes of the wild type mice only. There were few labeled cells in the transgenic lymph nodes. This may be due to alterations in the homing receptors of the T cells in the transgenic mouse lymph nodes. The percentages of CD4^+ ^and CD8^+ ^T cells in the non-transgenic recipient mouse lymph nodes were significantly higher than the transgenic mice when they received cells from immunized donor mice (Figure 7b). The proportion of CD8^+ ^T cells was higher than CD4^+ ^T cells in lymph nodes of these wild type recipients of immunized donor mice. There was no difference between the transgenic and non-transgenic recipient mouse groups when they received transfers from non-immunized donors. In contrast to wild-type mice, donor cells from immunized mice homed to the liver of transgenic mice as demonstrated by a three-fold increase in both CD4^+ ^and CD8^+ ^T cells compared to the other groups of recipient mice (Figure 8). This may indicate a trapping or homing mechanism for T-cells in transgenic mouse livers due to the dominant expression of the HCV transgene.

### Immunofluorescence analysis and histological changes in the livers of recipient mice

**Figure 9:**
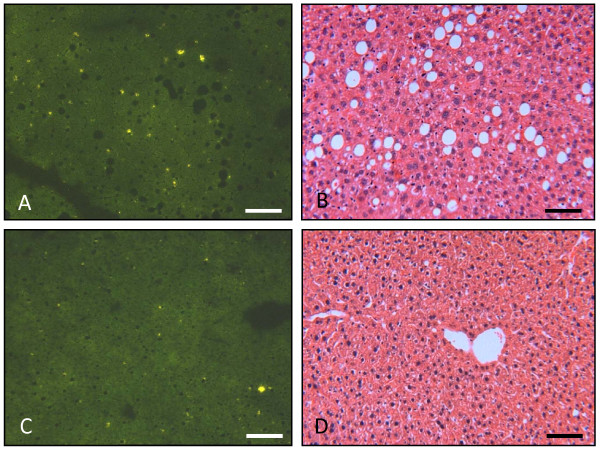
**Histological alterations in livers from transgenic and non-transgenic mice injected with CFSE-labeled splenocytes from immunized mice**. A) Immunofluorescent analysis of frozen liver sections (5 μm thick) of a transgenic mouse showing CFSE labeled cells scattered over all the liver section. The fluorescent cells are indicated by arrows. B) H&E stained liver section of transgenic mouse showing steatosis. There is infiltration of lymphocytes in the liver which is concentrated close to hepatic steatosis (indicated by arrows). C) Immunofluorescence analysis of frozen liver sections (5 μm thick) of non-transgenic mouse showing no CFSE labeled cells over the liver section. D) H&E staining of liver section of non-transgenic mouse showing normal histology of the liver and no lymphocyte infiltration. Scale bar = 50 μm.

**Figure 10:**
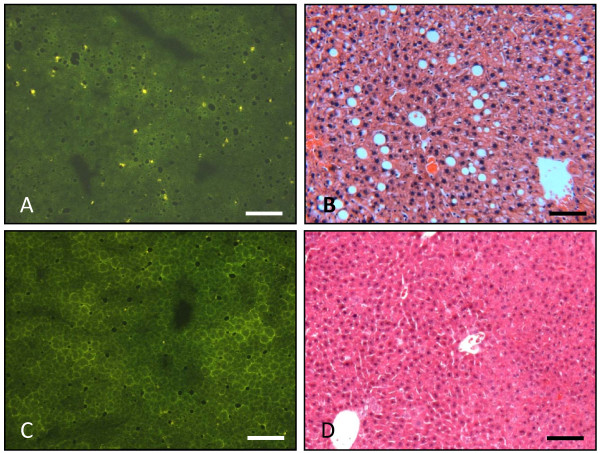
**Histological alterations in livers from transgenic and non-transgenic mice injected with CFSE-labeled splenocytes from non-immunized mice**. A) Immunofluorescent analysis of frozen liver sections (5 μm thick) of a transgenic mouse showing few CFSE labeled cells scattered over all the liver section. B) H&E stained liver section of transgenic mouse showing steatosis. There is no infiltration of lymphocytes in the liver. C) Immunofluorescence analysis of frozen liver sections (5 μm thick) of non-transgenic mouse showing no CFSE labeled cells over the liver section. D) H&E staining of liver sections of non-transgenic mouse showing normal histology of the liver and no lymphocyte infiltration. Scale bar = 50 μm.

Immunofluoresence analysis of the liver sections of the transgenic mice showed infiltration of high number of the CFSE labeled cells, when they received transfer from immunized mice (Figure 9a). H&E staining of the liver sections for the same group of recipient mice showed infiltration of lymphocytes beside the histological changes, such as steatosis, due to the expression of transgenes (Figure 9b). Interestingly, the infiltrated cells were concentrated in the areas where there was steatosis. On the other hand, the transgenic mice receiving cells from non-immunized donors showed few CFSE labeled cells on the liver sections and no cell infiltration was observed in the H&E stained liver section (Figure 10a, b). The non-transgenic mice showed no histological changes and no infiltration of CFSE labeled cells, whether they received donor cells from immunized (Figure 9c, d) or non-immunized mice (Figure 10c, d). Thus, repetitive transfer of splenocytes from HCV immunized mice into HCV transgenic mice may be needed in order to increase inflammation in the liver.

## Discussion

In our previous study, we showed an HCV transgenic mouse model expressing HCV structural proteins (core, E1 and E2) in the liver [17]. These transgenic mice developed liver steatosis, hepatopathy and tumor formation due to HCV protein expression. In this study, we describe an adoptive transfer from HCV immunized mice to HCV transgenic mice. As shown previously [18] as well as in this study, mice immunized with a combination of a candidate HCV vaccine consisting of recombinant HCV core/E1/E2 DNA plasmid, recombinant HCV polyprotein and montanide demonstrate a significant humoral and cellular antiviral immune responses. In order to confirm the specificity of the antiviral immune response and to assist the immune response mediated liver damage associated with hepatitis C infection, the splenocytes from the immunized mice were transferred to HCV transgenic mice. Seven days after the adoptive transfer, there was a significant decrease in the percentage of CFSE-labeled CD4^+ ^and CD8^+ ^T cells in the peripheral blood of transgenic mice that received cells from immunized donors, whereas the non-transgenic mice maintained a high percentage of the transferred T cells in their blood. This indicates that injected cells migrated from the peripheral blood and homed in different mouse organs. For instance, the number of CFSE labeled T cells from immunized mice was significantly higher in the liver of recipient transgenic mice as compared to those that received CFSE labeled T cells from non-immunized animals. T cells from HCV immunized mice that selectively homed in transgenic mouse livers, was likely due to the recognition of HCV transgenes or antigens which are preferentially expressed in this organ.

The immune responses against pathogens depend on the ability of lymphocytes to migrate to organs where the pathogen antigens exist. Here we have studied the kinetics of transferred lymphocytes in various organs of recipient mice. The lymphocytes derived from HCV immunized mice homed in HCV transgenic livers where the HCV antigens were predominantly expressed. In contrast, the lymphocytes from naïve mice homed in the spleen of non-transgenic recipient mice whereas lymphocytes from immunized donors homed preferentially in the non-transgenic recipient lymph nodes. Those cells are likely activated and perhaps recognize different homing receptors than lymphocytes from naive animals.

The CD4^+ ^and CD8^+ ^T cells from immunized mice frequently display activation markers. Although activated cells are more likely to migrate to the liver, more cells from immunized animals homed in this organ than cells from naïve animals, suggesting immune specificity against viral antigens. It was demonstrated that during adaptive immune responses two types of antigen-experienced T cells were produced; short-lived effector T cells, which would home to the sites where the pathogen was present, and long-lived memory T cells, that could provide protection against the pathogen they had encountered during the previous immune responses [19]. According to their findings, we hypothesized that lymphocytes from immunized mice would include both effector T cells that produced IFN-γ and memory T cells. However, more studies should be done to distinguish these in such immune response. Effector and memory T cells experienced with HCV antigens are the cells that more likely home to the transgenic livers. Another fraction of memory T cells stay in the lymph nodes. HCV-experienced or activated T cells homed in the lymph nodes of non-transgenic mice because there was no specific target in the non-transgenic donors. The increased knowledge on the mechanisms that regulate lymphocyte homing and imprinting has clear applications in designing more effective immunotherapeutic regimens.

There is strong evidence for the important role of both virus-specific CD4^+ ^and CD8^+ ^T cells in HCV virus clearance as well as in mediating liver cell damage in chronic hepatitis C infection [20,21]. The two major mechanisms of T-cell mediated lysis are perforin-granzyme-mediated cytotoxicity and Fas-mediated cytotoxicity. Both mechanisms can kill the infected cells directly or by bystander killing which were demonstrated to be important in hepatic injury [22]. The Fas-Fas ligand system is reported to be associated with the killing of the hepatocytes in patients infected chronically with hepatitis C virus. The expression of Fas ligand was up-regulated in the hepatocytes of patients with chronic hepatitis [23,24]. Liver-infiltrating lymphocytes express Fas ligand which will bind with the Fas receptor on the surface of hepatocytes and initiate Fas-mediated cell death [11,25]. In previous studies it has been shown that CD8^+ ^T cells can kill the targets *in vivo *by cytolysis mechanisms mediated by perforin and TNF-α [14] or required IFN-γ [15,22]. There are several experimental models of immune-mediated liver damage in chronic hepatitis. Adoptive transfer models using transgenic animals expressing HBV proteins in hepatocytes have been previously described [26,27]. These mice develop tolerance to virus-encoded proteins, but infusion of non-tolerant T cells will cause liver inflammation. Despite that some studies using *in vitro *systems showed that HCV structural, core and E2 proteins, were able to cause immunosuppression [28-30], there is no evidence showing that transgenic mice expressing HCV core, E1 and E2 proteins have global immunosuppression [31].

## Conclusions

We were able to adoptively transfer non-tolerant T cells into a transgenic mice expressing HCV transgene in hepatocytes. The transfer results in rapid and selective accumulation of the activated T cells in the liver of the transgenic mice but not in mouse spleen or lymph nodes. In this study we did not detect the fate of the transferred cells; nonetheless, it seems that these cells have the potential to have an antiviral effect that may result in liver inflammation and, subsequently a more severe injury.

Moreover, we suggest that performing adoptive transfer of splenocytes from HCV immunized mice into HCV transgenic mice may provide a good model to study the mechanisms of hepatic injury in chronic hepatitis C infection. This study may potentially help the development of an immunotherapeutic strategy for HCV infection.

## Competing interests

The authors declare that they have no competing interests.

## Authors' contributions

TN and MG have made substantial contributions to conception and design, acquisition of data, carried out the molecular genetic studies and drafted the manuscript. PG, CS and NS have carried out the immunoassays. RM participated in designing the study. FDM coordinated the study and helped to draft the manuscript. All authors read and approved the manuscript content.
